# Quantification of 1D, a novel derivative of curcumin with potential antitumor activity, in rat plasma by liquid chromatography-tandem mass spectrometry: application to a pharmacokinetic study in rats

**DOI:** 10.1080/13880209.2019.1603243

**Published:** 2019-04-24

**Authors:** Jialin Sun, Tao Jiang, Wen Xu, Zhangying Feng, Xianghua Quan, Ping Leng, Wei Sun, Jun Zhao, Fanbo Jing, Jing Li

**Affiliations:** a Department of Pharmacy, the Affiliated Hospital of Qingdao University, Qingdao, PR China;; b Key Laboratory of Marine Drugs Chinese Ministry of Education School of Medicine and Pharmacy, Ocean University of China, Qingdao, PR China;; c Department of Pharmacy, The Fourth Hospital of Hebei Medical University, Shijiazhuang, PR China

**Keywords:** Isothiouronium-modified pyrimidine-substituted curcumin, LC-MS/MS, intravenous

## Abstract

**Context:** 1 D is a novel derivative of curcumin and shows very promising antitumor activities in various cancer cell lines.

**Objective:** To characterize its preclinical pharmacokinetic profiles, a novel liquid chromatography-tandem mass spectrometry (LC-MS/MS) method was developed and validated for the quantification of 1 D in rat plasma.

**Materials and methods:** An aliquot of 50 μL plasma sample was processed by protein precipitation with methanol. Chromatographic separation was accomplished on a Zorbax Eclipse Plus C_18_ column (2.1 mm × 50 mm, 1.8 μm) with a gradient elution system (water/0.1% formic acid and methanol). Detection was performed by multiple reaction monitoring (MRM) mode using electrospray ionization in the positive ion mode. The optimized fragmentation transition for 1 D was *m/z* 491.2 → 361.2.

**Results:** The method was linear over the concentration range of 5–1000 ng/mL. The intra- and inter-day precisions were less than 9.8% and the accuracy was within ± 14.5%. The mean recovery of 1 D ranged from 102.5 to 105.9%. No matrix effects and significant sample loss during sample processing were observed. The validated method has been successfully applied to a pharmacokinetic study in rats after intravenous administration of 1 D. Non-compartmental pharmacokinetic parameters, including half-life (*t_1/2_*), apparent volume of distribution (V_z_), clearance (CL_z_), and area under the concentration-time curve (*AUC*_(0–t)_) were 4.92 h, 46.56 L/kg, 6.33 L/h/kg, and 806.70 μg/L/h, respectively.

**Discussion and conclusions:** Results demonstrated that 1 D displayed favourable pharmacokinetic properties for further *in vivo* pharmacologic evaluation, which could be facilitated by the validated LC-MS/MS method.

## Introduction

Curcumin is a natural polyphenol compound derived from turmeric [*Curcuma longa* Linn., (Zingiberaceae)]. It has been used widely in Ayurvedic medicine for centuries because it is non-toxic and has various therapeutic properties, including antioxidant (Altintoprak et al. 2016; Momeni and Eskandari 2017), analgesic (Jacob et al. 2013; Bulboaca et al. 2017), anti-inflammatory (Ma et al. 2017; Shakeri and Boskabady 2017), and antibiotic activities (Xie et al. 2015; Izui et al. 2016). Recently, a number of preclinical studies have demonstrated that curcumin has anticancer effects on a variety of tumours, including pancreatic (Bimonte et al. 2016), oesophageal (Lin et al. 2014), gastric (Barati et al. 2019), liver (Ren et al. 2018), lung (Liu et al. 2017), and uterine cancers (Li et al. 2013). Mechanism studies have found that it can participate in various biological pathways involved in apoptosis, tumour proliferation, chemo- and radiotherapy sensitization, tumour invasion, and metastases (Shehzad et al. 2013; Mehta et al. 2014; Su et al. 2017; Yang et al. 2017; Hurtado et al. 2018; Yu et al. 2018; Zhang et al. 2018). Although its advantages of safety, efficiency, and low toxicity, clinical applications of curcumin are restricted by its short half-life, low solubility, and poor stability (Anand et al. 2007; Zhou et al. 2014; Akbar et al. 2018). These inherent problems prompted us to synthesize novel curcumin analogues with better pharmacokinetic properties.

In the pursuit of safe and effective anti-tumour agents, we have designed and synthesized many curcumin derivatives (Qiu et al. 2013; Shen et al. 2015; Tong et al. 2016), among which, 1 D [(E,E)-4-(4,6-bis(4-methoxystyryl)pyrimidin-2-yloxy)butyl carbamimidothioate hydrobromide] has shown excellent antitumor activity (Tong et al. 2016). The IC_50_ values of 1 D treatment for 48 h in four human cancer cell lines were estimated to be 0.79 μM in HT29 cells, 1.00 μM in HCT116 cells, 0.92 μM in HJ1299 cells, and 0.99 μM in A549 cells, respectively, which indicated that 1 D had increased antitumor activity *in vitro* relative to curcumin. Based on its superior pharmacological activity, 1 D was selected as a drug candidate for treating tumours.

Although the pharmacological activity and mechanism of 1 D were studied in-depth, the pharmacokinetic (PK) properties were still unknown. It is well known that during the development of a new drug candidate, it is essential to obtain information regarding its pharmacokinetic parameters as early as possible (Baselga et al. 2012; US Food and Drug Administration [FDA] 2018). To further understand the pharmacokinetic characters of 1 D, a simple, rapid, and sensitive liquid chromatography-tandem mass spectrometry (LC-MS/MS) method was developed and validated in this study, and was applied to the pharmacokinetic study of 1 D in rats following single-intravenous administration. The method developed in this study will support and facilitate the design and selection of drug candidates with desirable pharmacokinetic properties. Moreover, our results will support optimization of dosing regimens for future preclinical efficacy studies.

## Materials and methods

### Reagents and chemicals

**Figure 1.**
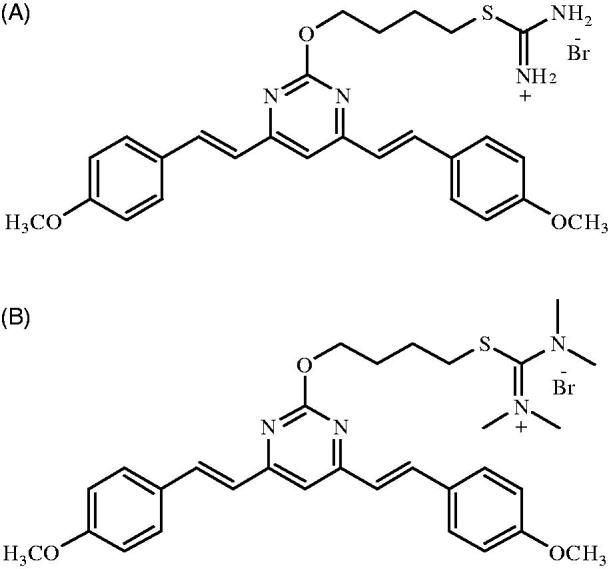
Chemical structures of 1 D (A) and IS (B).

1D (Figure 1(A), purity > 99%) and the internal standard (IS) 1 G [(E,E)-2-(4-(4,6-bis(4-methoxystyryl)pyrimidin-2-yloxy)butyl)-1,1,3,3-tetramethylisothiouronium hydrobromide] (Figure 1(B), purity > 99%) were synthesized and purified as previously described (Tong et al. 2016). LC-MS-grade methanol (MeOH) and HPLC-grade formic acid (HCOOH) were purchased from TEDIA (Fairfield, OH, USA). Analytical grade polyethylene glycol 400 (PEG400), poly (propylene glycol) 400 (PG400), and DMSO were obtained from Nanjing Chemical Reagent Co. (Nanjing, China). Ultra-pure water for the mobile phase was purified using a Milli-Q system (Millipore, Bedford, MA, USA). Blank plasma was purchased from Chundu Biotechnology Co., Ltd. (Wuhan, Hubei, China) and was stored at −80 °C.

### Instruments and analytical conditions

An AB ACIEX API 4000 triple-quadrupole mass spectrometer (Framingham, MA, USA) with electrospray ionization (ESI) interface was coupled with an Agilent 1290 Infinity II (Palo Alto, CA, USA) high performance liquid chromatography system consisting of a G7120A pump, a G4212-60008 inline degasser, a G7167B autosampler, and a G7116B column oven. Separation of the analyte and IS was achieved by using a Zorbax Eclipse Plus C_18_ column (2.1 mm × 50 mm, 1.8 μm) maintained at 40 °C. H_2_O (containing 0.1% HCOOH) (solvent A) and MeOH (solvent B) were used as gradient eluting mobile phases. The gradient was set as follows: 0 min 35% B, 1.5 min 35% B, 1.6 min 95% B, 3.5 min 95% B, 3.6 min 35% B, 5.0 min 35% B, then stopped. The flow rate was set at 0.4 mL/min and the injection volume was 2 μL.

The analytes were determined in positive ESI mode and quantified by multiple-reaction monitoring (MRM) mode. The source parameters were as follows: ion spray voltage, 5500 V; temperature, 550 °C; collision gas, 8 psi; curtain gas, 20 psi; ion source gas 1, 55 psi; and ion source gas 2, 55 psi, respectively. Compound dependent parameters which were optimized manually were as follows: entrance potential (EP), 10 V; cell exit potential (CEP), 13 V; collision energy (CE), 24 V for 1 D and 35 V for IS; and declustering potential (DP), 50 V for 1 D and 60 V for IS, respectively.

**Figure 2.**
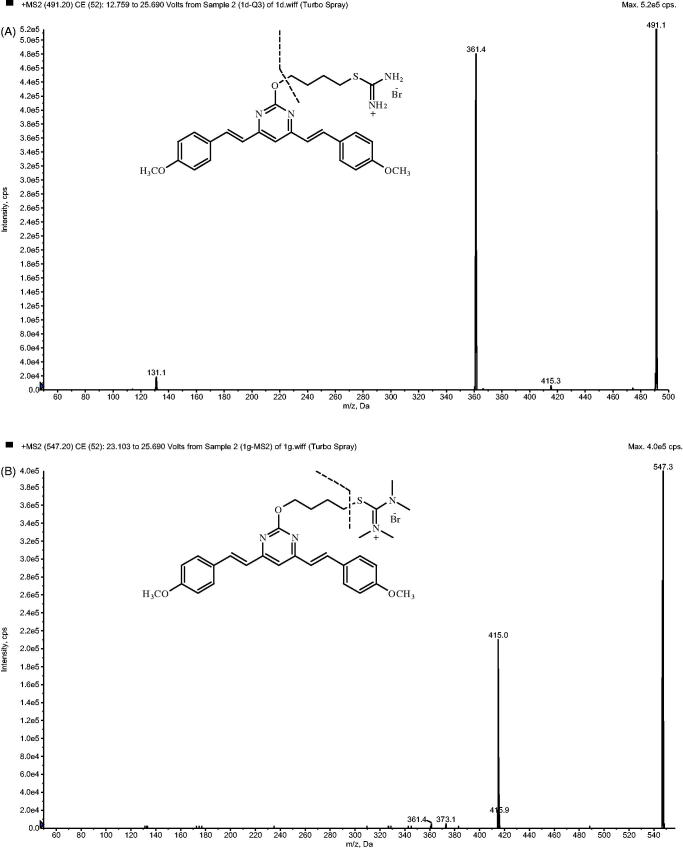
Product ion mass spectrum of 1 D (A) and IS (B).

Quantification was performed using MRM mode. The transitions were *m/z* 491.2 → 361.2 for 1 D and 547.2 → 415.1 for IS (Figure 2), respectively. All the operations, acquiring and analysis of data, were controlled by AB Sciex Analyst software (version 1.6.3).

### Stock and working solution preparation

Stock solutions of 1 D and IS at a concentration of 500 μg/mL were prepared by dissolving accurate amounts of reference standards in MeOH. Stock solutions were further diluted with MeOH to get a series of working solutions at the concentrations of 50, 100, 200, 500, 1000, 2000, 5000, and 10000 ng/mL for 1 D and 4000 ng/mL for IS. All stock and working solutions were stored at −20 °C and brought to room temperature before use.

Plasma samples for calibration were prepared by spiking 5 μL of corresponding 1 D working solutions into blank plasma to obtain final concentration levels of 5, 10, 20, 50, 100, 200, 500, and 1000 ng/mL. Quality control (QC) samples of 5, 10, 100, and 500 ng/mL were prepared similarly.

### Plasma sample preparation

IS working solution (5 μL) and 95 μL of MeOH were added to 50 μL of plasma sample in a 1.5 mL centrifuge tube. The mixture was then vortexed for 3 min and centrifuged at 13,400 rpm for 10 min. A 2 μL aliquot of the supernatant was injected for LC-MS/MS analysis.

### Method validation

A full method validation was performed according to US FDA guidelines (FDA 2018) with respect to specificity, linearity, precision, accuracy, recovery, matrix effect, and stability.

### Specificity

The LC-MS/MS method specificity was evaluated by comparing blank plasma from six individual rats with corresponding spiked plasma samples and plasma samples from rats after intravenous administration to exclude interference of endogenous substances.

### Linearity and sensitivity

An eight-point standard calibration curve over the concentration range of 5–1000 ng/mL was constructed by using the peak area ratio (*y*) of 1 D to the IS versus concentration (*x*). Each calibration curve was performed individually by using least square weighted (1/*x*^2^) linear regression. As the minimum standard of the calibration curve, the lower limit of quantification (LLOQ) should have an analyte response at least 5 times the blank response. The calibration curve was acceptable if the correlation coefficient (*r*) value was greater than 0.99 and the back-calculated concentration had a precision that did not exceed 15% of the relative standard deviation (RSD) and accuracy was within 15% of the nominal concentration (20% for LLOQ).

### Precision and accuracy

The intra- and inter-day accuracy and precision of 1 D were performed by analyzing five replicates of QC samples (5, 10, 100, and 500 ng/mL) on the same day and on three consecutive days. The precision was expressed as RSD and accuracy was expressed as the deviation of the mean from the nominal value. Acceptable criteria included precision within 15% RSD and accuracy within ± 15%.

### Recovery and matrix effect

Recovery and matrix effect were analyzed at four QC concentrations (5, 10, 100, and 500 ng/mL). The recovery was investigated by comparing the responses of the analytes in blank matrix samples (analytes spiked prior to deproteinization) with those in post-extracted blank plasma samples (analytes added after deproteinization) (*n* = 5). The matrix effect was calculated by comparing the responses of the analytes in post-extracted blank plasma samples with those of corresponding standard solutions (*n* = 5).

### Stability

The stability of 1 D in rat plasma was evaluated in five replicates at three QC levels under various storage conditions. These results were compared with those obtained from freshly prepared samples to calculate the percentages of intact drug remaining. Short-term and long-term stability were evaluated by storing plasma samples at room temperature for 6 h and −20 °C for 1 month before sample preparation. Freeze-thaw stability of QC samples was assessed after three freeze-thaw cycles (−20 to 25 °C). Autosampler stability was evaluated by keeping the extracted plasma samples in the autosampler for 24 h before analysis. Samples were considered to be stable if assay values were within the acceptable limits of accuracy (±15%) and precision (15% RSD).

### Pharmacokinetic studies and data analysis

The applicability of the developed bioanalytical LC-MS/MS method was evaluated by quantitative determination of 1 D in rat plasma after single intravenous administration. All experimental procedures and animal care were performed following approval from the Ethic Committee of Laboratory Animals of the Affiliated Hospital of Qingdao University. Six male Sprague–Dawley rats, weighing 200 ± 15 g, were obtained from Beijing Vital River Experimental Animal Co. Ltd (Beijing, China). The animals were quarantined for 1 week prior to the study and were maintained on a 12 h light/12 h dark cycle at 22 ± 2 °C and at 50 ± 10% relative humidity. The rodents were given a commercial rat chow and water *ad libitum*. An intravenous dose of 5 mg/kg was selected based on our previous toxicological and pharmacodynamic studies in rats (unpublished data). The administration solution was prepared by dissolving 1 D in PEG400: PG400: DMSO: saline (30: 30: 1: 39, v/v/v/v) to give a concentration of 2 mg/mL. After fasting overnight, rats were given the prepared administration solution at 5 mg/kg via the tail vein. Blood samples (0.2 mL) were collected into heparinized tubes by retro-orbital puncture at 0 (pre-dose), 5, 10, 20, 30, and 45 min, and 1, 1.5, 2, 4, 8, 12, and 24 h after dosing. Plasma was separated by centrifugation at 4500 rpm for 10 min and stored at −20 °C until analysis.

The pharmacokinetic parameters including area under the concentration-time curve (AUC), apparent volume of distribution (V_z_), elimination half-life (*t_1/2_*), and clearance (CL_z_) were calculated by a non-compartmental analysis method using the DAS 3.0.2 pharmacokinetic programme. As observable parameters, the maximum plasma concentration (*C_max_*) and the time to reach the maximum concentration (*T_max_*) were obtained directly.

## Results and discussion

### LC-MS/MS optimization

The MS intensity of 1 D and IS was optimized after infusion of each standard solution into the mass spectrometer. The signal intensity of the compounds was found to be greater in the positive mode after testing each compound in both positive and negative modes. The protonated molecular ion [M + H]^+^ was chosen as the parent ion in the Q1 full scan spectra for 1 D at *m/z* 491.2 and for IS at *m/z* 547.2. The parent ion was used as the precursor ion to obtain the production in the Q3 spectra. The fragmentations with the highest relative abundance were at *m/z* 361.2 and 451.1 for 1 D and IS, respectively. Therefore, quantification was performed using MRM mode and the transitions were *m/z* 491.2 → 361.2 for 1 D and *m/z* 547.2 → 415.1 for IS. The MS parameters, such as DP and CE, were optimized to ensure that MRM transitions were sensitive.

Various mobile phases with different concentrations of acetonitrile (ACN), MeOH and HCOOH were tested to obtain short run times, well-resolved peaks, and peaks with symmetrical shapes. Finally, high response, good separation, and superior sensitivity of the analyte peaks were obtained by using a gradient eluted mobile phase consisting of MeOH and H_2_O (containing 0.1% HCOOH).

### Internal standard selection

An appropriate IS is important to ensure the reproducibility and accuracy of LC-MS/MS method by adjusting the deviations derived from operational and/or instrumental errors. The chromatographic behaviour, recovery, and ionization properties of IS should be similar to the analytes. Analogues of the analytes are often applied as IS. In this study, the pyrimidine-substituted curcumin analogue 1 G, which was the homologue of 1 D, was selected as IS based on their consistent recovery and the non-interfering molecular ion peaks.

### Extraction process optimization

Due to the complex nature of plasma, pre-treatment of plasma samples is an important step in removing proteins and potential interference prior to LC-MS/MS analysis. Different extraction conditions were investigated, including protein precipitation by MeOH or ACN, and liquid-liquid extraction by ethyl acetate. Results showed that the sensitivity and specificity of these methods could all meet the research requirements. Compared to the liquid-liquid extraction method, extraction procedure of protein precipitation required less time and could decrease the cost of the assay. Finally, MeOH was selected as the precipitation solvent because it was compatible with the mobile phase and could avoid asymmetric and spread-out peak shapes.

### Method validation

#### Specificity

**Figure 3.**
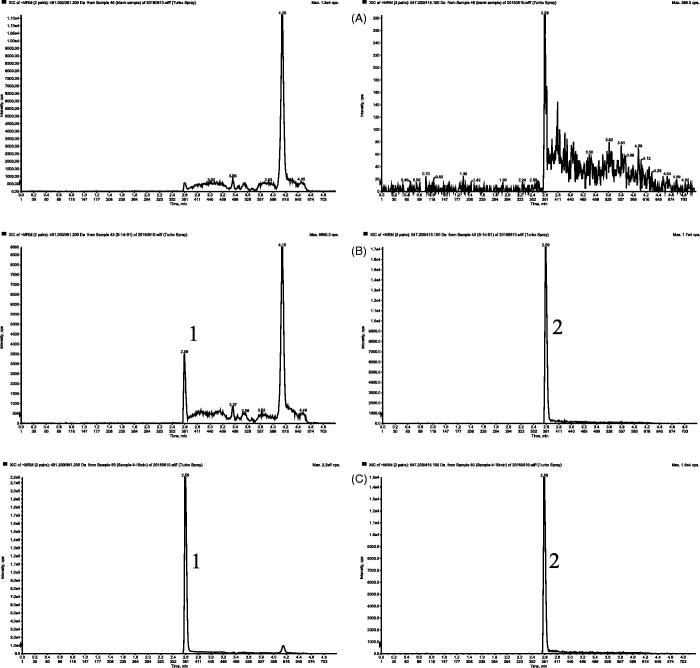
Representative MRM chromatograms of 1 D (1) and IS (2) in rat plasma samples: (A) blank plasma sample; (B) blank plasma sample spiked with 1 D (5 ng/mL, LLOQ) and IS (400 ng/mL); and (C) plasma sample at 10 min after an intravenous administration of 5 mg/kg 1D.

The typical chromatograms of blank plasma, blank plasma spiked with 1 D and IS, and plasma sample obtained 10 min after intravenous administration of 1 D are presented in Figure 3. No significant interference from blank rat plasma was observed at the retention time of the analytes. The retention time of 1 D and IS was approximately 3.8 min.

#### Linearity and sensitivity

Good linearity was obtained over the eight concentrations ranging from 5 to 1000 ng/mL for 1 D. The regression equation was *y =* 0.005961*x* + 0.010111 (*r* = 0.998). All standards were deviated by less than 10.6% of nominal concentrations. The LLOQ was 5 ng/mL with a precision of 4.5% and an accuracy of 96.4% (*n =* 5).

#### Precision and accuracy

Table 1 shows the results of the intra- and inter-day precision and accuracy for 1 D determined by the QC samples at four concentration levels (5, 10, 100, and 500 ng/mL) with five replicates at each level. The accuracy was assessed by calculating the recovery relative to the nominal value, which was between 86.1–114.5%. The values for intra- and inter-day precision were less than 9.8% in all cases. All the results were within the acceptable criteria.

**Table 1.** Intra- and inter-day precision and accuracy of the method for 1 D in rat plasma (*n* = 5).

	Spiked concentration (ng/mL)	Measured concentration (mean ± SD, ng/mL)	RSD (%)	Accuracy (%)
intra-day	5	4.84 ± 0 .38	7.9	96.8
	10	10.26 ± 0 .61	6.0	102.6
	100	105.71 ± 7.99	7.6	105.7
	500	493.10 ± 48.11	9.8	98.6
inter-day	5	4.94 ± 0.33	6.7	98.8
	10	10.15 ± 0.73	7.2	101.5
	100	99.74 ± 7.83	7.8	99.7
	500	497.14 ± 30.24	6.1	99.4

#### Recovery and matrix effect

As shown in Table 2, the extraction recovery of 1 D was in the range of 102.5–105.9% at four concentration levels and the matrix effects of the four QC concentration samples ranged from 91.3–97.5%. The extraction recovery and matrix effect of IS were calculated to be 108.4 ± 4.9% and 91.0 ± 32.7%, respectively. No significant matrix effects on the analytes were observed. Results indicated that the extent of recovery of 1 D and IS was consistent and reproducible, no co-elution substances affected the ionization of the analytes, and the protein precipitation efficiency was satisfactory.

**Table 2.** Recovery and matrix effect of the method for 1 D and IS in rat plasma (*n* = 5).

Compd.	Spiked concentration (ng/mL)	Recovery		Matrix effect	
		mean ± SD (%)	RSD (%)	mean ± SD (%)	RSD (%)
1D	5	103.7 ± 6.0	5.8	97.3 ± 6.3	6.5
	10	105.9 ± 10.6	10.0	97.5 ± 9.7	9.9
	100	102.5 ± 6.9	6.8	91.3 ± 0.8	0.9
	500	103.5 ± 7.7	7.4	95.3 ± 2.6	2.8
IS	400	108.4 ± 4.9	4.5	91.0 ± 2.7	3.0

#### Stability

Stability experiments were performed and the results are summarized in Table 3. No significant changes in concentrations of 1 D were measured after storage at room temperature for 6 h, storage at −20 °C for 1 month, storage in autosampler for 24 h, and three freeze-thaw cycles. The data confirmed the stability of 1 D in rat plasma during the storage and sample processing.

**Table 3.** Stability of the method for 1 D in rat plasma (*n* = 5).

Spiked concentration (ng/mL)	Condition	Measured concentration (mean ± SD, ng/mL)	RSD (%)	Accuracy (%)
10	Control	10.34 ± 0.7	6.8	103.4
	6 h at 25 °C	10.27 ± 0.56	5.5	102.7
	4 weeks at −20 °C	10.61 ± 0.55	5.2	106.1
	Three freeze-thaw cycles	10.12 ± 0.66	6.5	101.2
	Autosampler for 24 h	10.36 ± 0.75	7.2	103.6
100	Control	100.27 ± 7.02	7.0	100.3
	6 h at 25 °C	104.71 ± 5.16	4.9	104.7
	4 weeks at −20 °C	98.39 ± 7.52	7.6	98.4
	Three freeze-thaw cycles	99.36 ± 6.92	7.0	99.4
	Autosampler for 24 h	101.51 ± 6.43	6.3	101.5
500	Control	520.75 ± 35.48	6.8	104.2
	6 h at 25 °C	530.28 ± 34.32	6.5	106.1
	4 weeks at −20 °C	518.57 ± 43.30	8.4	103.7
	Three freeze-thaw cycles	503.88 ± 33.79	6.7	100.8
	Autosampler for 24 h	481.19 ± 42.98	8.9	96.2

### Application of the method

**Figure 4.**
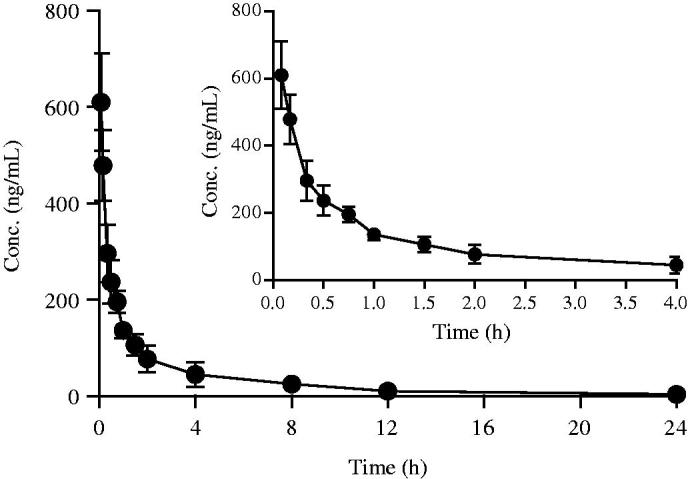
Mean concentration-time curves of 1 D following single-dose intravenous administration at 5 mg/kg in rats (mean ± SD, *n* = 6).

The validated method was successfully applied to study the pharmacokinetic behaviours of 1 D in rats following a single intravenous administration at 5 mg/kg. The plasma concentration-time profile is presented in Figure 4. The typical pharmacokinetic parameters from a non-compartmental model analysis were as follows: AUC_(0-t)_=806.70 ± 259.05 ug/L·h, *t_1/2_* =4.92 ± 2.00 h, CL_z_ = 6.33 ± 2.04 L/h/kg, V_z_ = 46.56 ± 27.32 L/kg. The results are summarized in Table 4. The results properly indicated that moderate clearance and high extravascular distribution would be exhibited by 1 D *in vivo*.

**Table 4.** Pharmacokinetic parameters of 1 D following single-dose intravenous administration at 5 mg/kg in rats (*n* = 6).

Parameters	Unit	Intravenous	
		Mean	SD
AUC_(0−t)_	ug/L·h	806.70	259.05
AUC_(0−∞)_	ug/L·h	852.60	243.07
MRT_(0−t)_	h	3.40	1.20
MRT_(0−∞)_	h	4.70	1.22
*t_1/2_*	h	4.92	2.00
*T_max_*	h	0.08	0.00
V_z_	L/kg	46.56	27.32
CL_z_	L/h/kg	6.33	2.04
*C_max_*	ug/L	610.17	100.75

AUC_(0−t)_: area under the curve from time zero to the last sampling time point; AUC_(0−∞)_: area under the curve from time zero to the infinity; MRT_(0−t)_: mean residence time from time zero to the last sampling time point; MRT_(0−∞)_: mean residence time from time zero to the infinity; *t_1/2_:* elimination half-life; *T_max_*: time of maximum concentration; V_z_: apparent volume of distribution; CL_z_: clearance; *C_max_*: maximum concentration

## Conclusions

In this study, a sensitive, rapid, and reproducible LC-MS/MS method for the quantification of 1 D was developed and fully validated in rat plasma. Advantages of this method included a small blood sample amount requirement, sufficient recovery rate by a simple single-step protein precipitation, and a short running time for high throughput analysis. The present method was successfully applied to a pharmacokinetic study of 1 D in rats, and could probably be used to determine the concentration of 1 D in other biological matrices in future ADMET studies. The results of this study indicated that 1 D possessed favourable *in vivo* pharmacokinetic characteristics, including moderate clearance and high extravascular distribution, providing valuable information for its further development as a novel antitumor agent. As a promising preclinical candidate for the treatment of tumours, more research should be done on 1 D in the future.
